# Hodgkin's lymphoma presenting with markedly elevated IgE: a case report

**DOI:** 10.1186/1710-1492-5-12

**Published:** 2009-12-07

**Authors:** Anne K Ellis, Susan Waserman

**Affiliations:** 1 Division of Clinical Immunology & Allergy, Department of Medicine, McMaster University, Hamilton, ON, Canada; 2 Division of Allergy & Immunology, Department of Medicine, Queen's University, Kingston, ON, Canada

## Abstract

**Background:**

Markedly elevated IgE as a manifestation of a lymphoproliferative disorder has been only rarely reported.

**Case Presentation:**

We present the case of a 22 year old female referred to the adult Allergy & Clinical Immunology clinic for an extremely elevated IgE level, eventually diagnosed with Hodgkin's lymphoma. She had no history of atopy, recurrent infections, eczema or periodontal disease; stool was negative for ova & parasites. Chest X-ray revealed large bilateral anterior mediastinal masses that demonstrated prominent uptake on gallium scan. Mediastinal lymph node biopsy was consistent with Hodgkin's lymphoma, nodular sclerosing subtype, grade I/II.

**Conclusion:**

Although uncommon, markedly elevated IgE may be a manifestation of a malignant process, most notably both Hodgkin's and Non-Hodgkin's lymphomas. This diagnosis should be considered in evaluating an otherwise unexplained elevation of IgE.

## Background

Elevated levels of total serum IgE are associated with many diseases, including allergic bronchopulmonary aspergillosis (ABPA), parasitosis, atopic dermatitis, adult HIV infection, hyper-IgE (Job's) syndrome, Sézary's syndrome, IgE myeloma, and Kimura's disease[1]. Lymphoproliferative disorders are known associations of the hyper-IgE syndrome [2-4], however a marked elevation of IgE as an initial manifestation of a lymphoproliferative disease is rare, and mainly reported in IgE producing plasmacytomas; also rare (0.01% of plasmacytomas)[5]. Three cases are reported in the literature of non-Hodgkin's lymphoma associated with markedly elevated levels of IgE [6-8], one of which was asymptomatic and discovered serendipitously during an evaluation of perennial rhinitis[6]. Here we present a patient referred for evaluation of a markedly elevated IgE, eventually diagnosed with Hodgkin's lymphoma.

## Case Presentation

A 22 year old female was referred to our allergy clinic for evaluation of an elevated IgE in the setting of a 4 year history of fatigue; diffuse pruritus and a microcytic anemia (see Table). She denied weight loss, fever, or decreased appetite. She had night sweats while taking venlafaxine for depression, which resolved upon discontinuation of this medication. She had been diagnosed by Hematology with both B_12 _deficiency and a possible iron deficiency (serum Fe was low but ferritin and total iron binding capacity were normal (see Table); however, treatment with B12 injections and iron replacement did not correct the anemia. Bone marrow aspiration confirmed the presence of iron stores. There was associated thrombocytosis (platelet count 592 × 10^9^/L, reticulocytosis (retic count 100 × 10^9^/L), elevated C-reactive protein (146.0 mg/L) and an ESR of 50 mm/hr. Quantitative immunoglobulins demonstrated an IgE level of 22,562 kU/L, prompting the referral to Allergy & Immunology. Details of her investigations are summarized in Table 1.

**Table 1:** Laboratory parameters upon referral to Allergy & Immunology Clinic.

Parameter	Value	Reference	(Units)	Parameter	Value	Reference	(Units)
Creatinine	64	50-100	umol/L	WBC	10.2	4.0-11.0	×10^9/L

Urea	2.3	3.0-6.5	umol/L	Eosinophils	0.1	0.0-0.4	×10^9/L

Sodium	140	135-145	mmol/L	Hb	**103**	115-165	g/L

Potassium	3.7	3.5-5.0	mmol/L	MCV	**76.7**	82-99	fL

Chloride	104	98-107	mmol/L	Platelet	**592**	150-400	×10^9/L

Total Protein	**81**	60-80	g/L	Retic	**100**	10-86	×10^9/L

Albumin	**33**	35-50	g/L	ESR	**50**	1-20	mm/hr

A/G ratio	**0.7**	1.4-1.6		CRP	**122**	<3.0	mg/L

AST	14	<35	U/L	C3	1.67	0.73-1.73	g/L

ALT	22	<28	U/L	C4	0.3	0.13-0.52	g/L

GGT	**65**	<32	U/L	IgA	1.6	0.70-3.52	g/L

Alk Phos	**293**	40-120	U/L	IgD	4	<140	mg/L

Bilirubin	5	2-18	umol/L	IgE	**18 429**	<120	kU/L

Ferritin	173	51-400	ug/L	IgG	13.9	6.35-14.65	g/L

CK	27	<150	U/L	IgM	1.07	0.41-2.07	g/L

LDH	**308**	100-220	U/L	RF	<11.0	0-15.0	IU/mL

TIBC	43	4-80	umol/L				

Fe	**4**	9-30	umol/L				

She had no history of recurrent infections, eczema or periodontal disease, nor was there a history of foreign travel, diarrhea or other symptoms suggestive of parasitic infection. There was no history of allergic rhinitis (seasonal or perennial), asthma, sinusitis, otitis or other allergic disease. Her physical examination was entirely normal. Skin tests were positive to trees, grass and ragweed, and careful questioning confirmed an absence of clinical symptoms aside from intermittent cough. Stool examination was negative for ova & parasites. Spirometry and methacholine challenge revealed a mild isolated decrease in diffusion capacity, and no airway hyper-responsiveness.

**Figure 1:**
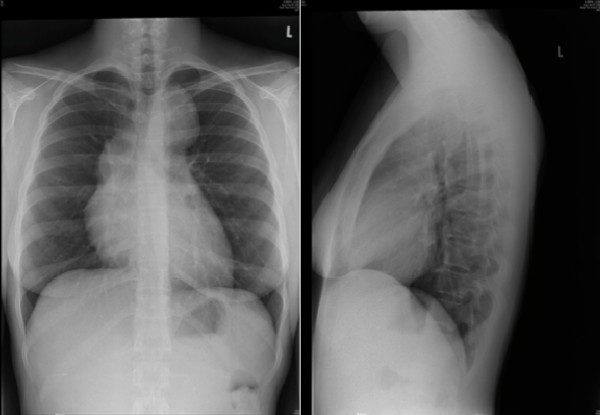
**Chest x-ray, PA and Lateral views**.

After initial investigations were completed, her symptomatology remained unexplained. Investigation was extended with repeat stool examination, and a chest x-ray, which revealed large bilateral anterior mediastinal masses (see Figure 1). Further evaluation with gallium scan demonstrated prominent diffuse uptake within these lesions, and a CT of the chest & abdomen confirmed the presence of multiple enlarged anterior mediastinal lymph nodes and mild hepatomegaly. A mediastinal lymph node biopsy was consistent with Hodgkin's lymphoma, nodular sclerosing subtype, grade I/II. She was reassessed by Hematology and treatment with ABVD (adriamycin, bleomycin, vinblastine and dacarbazine) was initiated. Ongoing treatment with ABVD has resulted in a partial response based on PET scan FDG (F-18 fluorodeoxyglucose) uptake; IgE has decreased to 4,014 kU/L.

## Discussion

Significant elevations of IgE are seen in various allergic conditions, parasitosis, and rarely, in lymphoproliferative malignancies. Specifically, extreme elevations of IgE have been documented in the setting of multiple myeloma, and B-cell lymphomas. In this case, the patient had no history of atopy, or parasitic infection and she had a normal protein electrophoresis and bone marrow evaluation.

Lymphomas are known to produce immunoglobulins, and rarely, cases have been reported of both B- and T-cell lymphomas associated with elevated IgE [6-8]. Sézary's syndrome (a peripheral T-cell neoplasm) has been associated with elevated IgE and/or eosinophilia when the malignant clone is of the CD4+ helper phenotype and produces an abnormal amount of the cytokine IL-4[9,10]. Modestly elevated IgE has also been reported in B-cell chronic lymphocytic leukemia[11] and in 2 patients with Hodgkin's disease (1 case of nodular sclerosing, one case of mixed cellularity, levels were 675 IU/mL and 310 IU/mL, respectively)[12].

## Conclusion

Markedly elevated IgE may rarely present as an initial manifestation of a lymphoproliferative disorder such as a lymphoma. These patients may be referred for evaluation of allergy or immunodeficiency, such as hyperIgE syndrome. This patient had unexplained fatigue and anemia, and only chest X-ray was suggestive of a malignant process. Underlying lymphoproliferative disease should always be considered when evaluating an otherwise unexplained significant elevation of IgE, particularly when features of allergy or parasitosis are distinctly lacking. Specific work-up of significantly elevated IgE levels should be tailored to the clinical features of the case, but in this circumstance a serum LDH and a CXR helped to reveal the underlying causative lymphoma.

## Consent

Written informed consent was obtained from the patient for publication of this case report and accompanying images. A copy of the written consent is available for review by the Editor-in-Chief of this journal.

## Comepeting interests

The authors declare that they have no competing interests.

## Authors' contributions

AKE and SW both saw this patient in an outpatient Allergy/Immunology clinic. AKE wrote the first draft of the manuscript and SW and AKE jointly worked on several subsequent revisions to the manuscript. Both AKE and SW contributed to the comments raised upon peer review and the final revised, accepted version of the manuscript. Both authors have read and approved the final manuscript.
